# Comparison of the Performance of Cartomizer Style Electronic Cigarettes from Major Tobacco and Independent Manufacturers

**DOI:** 10.1371/journal.pone.0149251

**Published:** 2016-02-18

**Authors:** Monique Williams, Amanda Villarreal, Barbara Davis, Prue Talbot

**Affiliations:** Department of Cell Biology and Neuroscience, University of California Riverside, Riverside, California, United States of America; Georgia Regents University, UNITED STATES

## Abstract

**Objective:**

This study compared the performance of 12 brands of cartomizer style electronic cigarettes (EC) using different puffing protocols and measured the concentrations of nicotine in each product.

**Methods:**

Air flow rate, pressure drop, and aerosol absorbance were measured using two different protocols, first 10 puffs and a modified smoke-out protocol.

**Results:**

First 10 puff protocol: The air flow rate required to produce aerosol ranged between brands from 4–21 mL/s. Pressure drop was relatively stable within a brand but ranged between brands from 14–71 mmH_2_O and was much lower than the earlier classic 3-piece models. Absorbance, a measure of aerosol density, was relatively consistent between puffs, but varied between brands. With the modified smoke-out protocol, most brands were puffed until 300 puffs. The pressure drop was relatively stable for all brands except three. Absorbance of the aerosol decreased as the number of puffs increased. Although there was some uniformity in performance within some brands, there was large variation between brands. The labeled and measured nicotine concentrations were within 10% of each other in only 1 out of 10 brands.

**Conclusions:**

Over 10 puffs, the cartomizers all perform similarly within a brand but varied between brands. In smoke-out trials, most brands lasted at least 300 puffs, and performed similarly within brands with respect to pressure drop and absorbance. For five brands, products purchased at different times performed differently. These data show some improvement in performance during evolution of these products, but nevertheless indicate problems with quality control in manufacture.

## Introduction

The original electronic cigarettes (EC) were three piece models, which had a separate battery, atomizing unit, and a cartridge for holding a fluid that usually contained propylene glycol and/or glycerol, flavoring chemicals, and nicotine [1–3]. In 2009, manufacturers combined the atomizer and cartridge into a single replaceable unit called a cartomizer. Cartomizer style ECs, which are currently the dominant marketed model in the USA, are readily available in supermarkets, drug stores, convenience stores, gas stations, and on the Internet. Cartomizers come in different flavors (e.g., tobacco, menthol, and coffee) and nicotine concentrations ranging from 0–36 mg/mL [4]. Major tobacco companies entered the EC market with cartomizers style EC in 2013. Many users refill them when the fluid runs low, and there are 1000s of refill fluids present on the market [4–7]. Since EC do not burn tobacco and contain fewer chemicals than conventional cigarettes, they are sometimes considered safer by advocates and consumers [8,9]. However, there are relatively few studies evaluating the health effects caused by EC use [10–12], and there is concern that some components in EC aerosol may be harmful [6,13–15].

In an earlier study, we compared the performance of the classic and cartomizer style EC [16]. The two cartomizer brands, Smoking Everywhere Platinum and Crown 7 Imperial, behaved similarly within brands, but varied between brands [16]. Crown 7 Imperial cartomizers were able to produce aerosol for 400 ± 10 puffs, in contrast to Smoking Everywhere Platinum which lasted 160 ± 66 puffs [16]. As was seen in the classic models, as the cartomizer EC were puffed, there was an increase in pressure drop and a decrease in absorbance [2,16]. This variability within and between non-disposable EC brands has been seen in the concentration of nicotine delivered to the consumer [17].

The purpose of the current study was to compare the performance of a broad range of cartomizer style EC from major tobacco and independent manufactures. Both short and long term puffing protocols were used to examine performance. The concentrations of nicotine in cartomizer style EC was also determined and compared to label values.

## Materials and Methods

### Electronic Cigarette Selection

**Fig 1:**
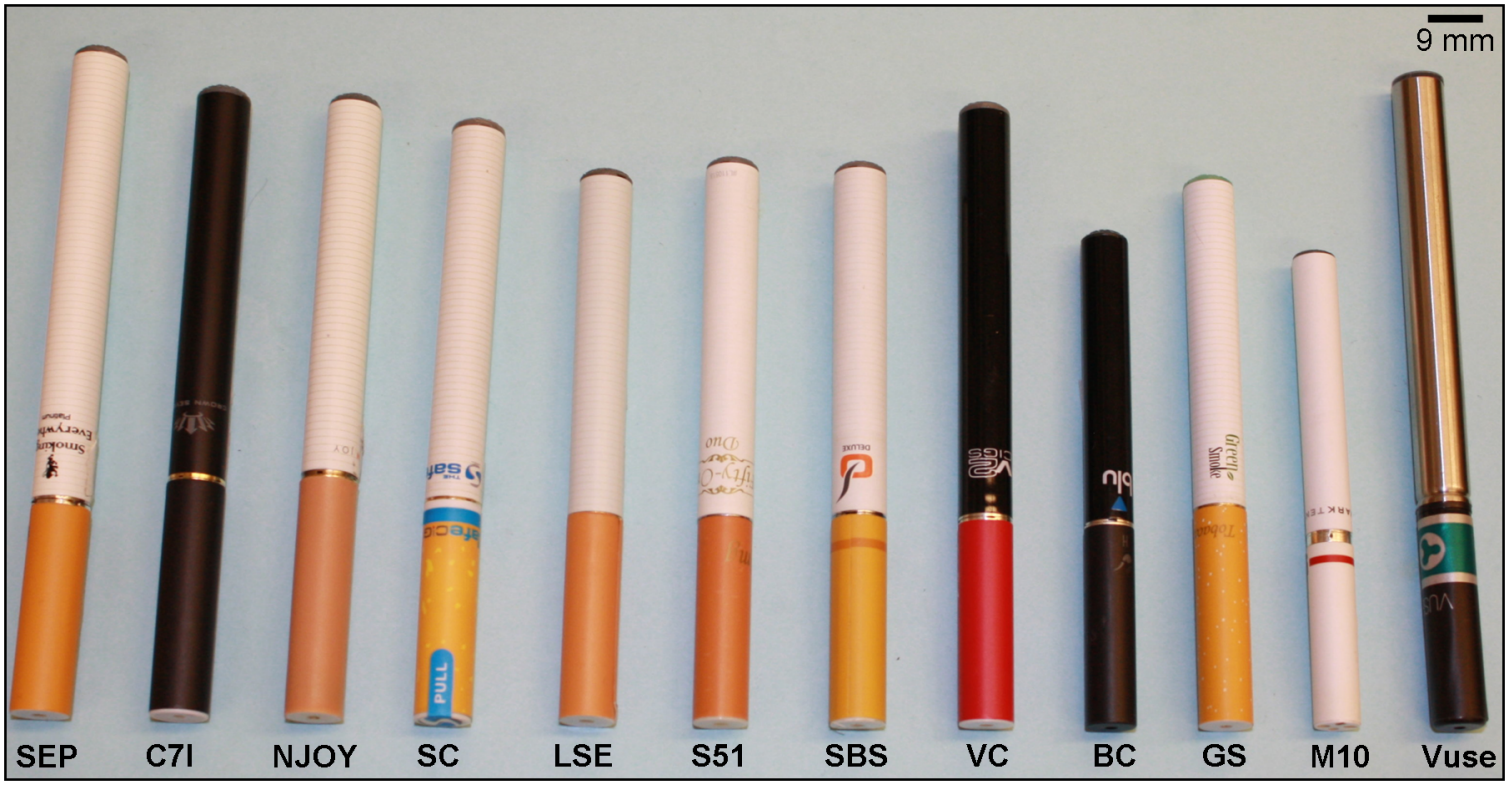
Cartomizer style EC used in this study. From Left to right: Smoking Everywhere Platinum (SEP), Crown 7 Imperial (C7I), NJOY NPRO (NJOY), SafeCig (SC), Liberty Stix Eagle (LSE), Smoke 51 (S51), South Beach Smoke (SBS), V2 Cigs (VC), BluCig (BC), Greensmoke (GS), Mark Ten (M10) and Vuse.

All EC were second generation cartomizer style models that were selected based on consumer reviews (Table 1). Brands selected for this study were: Smoking Everywhere Platinum (Smoking Everywhere, Sunrise, FL), Crown 7 Imperial Hydro (Crown Seven Shop, Scottsdale, AZ), NJOY NPRO 2N1 (Sottera Inc., Scottsdale, AZ), Safe Cig (The Safe Cig LLC, Los Angeles, CA), Liberty Stix Eagle (Liberty Stix, LLC, Cleveland, OH), Smoke 51 (Vapor Corp, Miami, FL), South Beach Smoke (South Beach Java LP, Wood Dale, IL), V2 Cigs (VMR Products LLC.), BluCig (Lorillard Inc., Greensboro, NC), Green Smoke (Green Smoke LLC, Richmond, VA), Mark Ten (Nu Mark LLC, Miami, FL) and Vuse (RJ Reynolds Vapor, Winston-Salem, NC) (Fig 1, Table 1).

**Table 1:** Performance Properties of Electronic Cigarettes.

Brand	Nicotine conc (mg)	Meas. conc (mg/mL)	% change	Avg Air Flow Rate (mL/s)			Avg Pressure Drop (mmH_2_O)			Avg Absorbance			Avg # Puffs	Consumer Rating
				1^st^ 10	1^st^ 10—SO	Smoke Out	1^st^ 10	1^st^ 10—SO	Smoke Out	1st 10	1^st^ 10—SO	Smoke out		
SE Platinum^a^^,^^b^	0	ND	NA	21 ± 0	20 ± 1	21 ± 1	68 ± 9	66 ± 8	79 ± 5	0.27 ± 0.15	0.26 ± 0.10	0.16 ± 0.05	160 ± 66	Highly Rated^c^
C7 Imperial^a^	High	13.54 ± 0.05	NA	9 ± 2	9± 2	15 ± 1	29 ± 13	20 ± 8	62 ± 8	0.24 ± 0.13	0.37 ± 0.07	0.21 ± 0.06	400 ± 10	Not Rated
NJOY NPRO	18	12.00 ± 0.12	-33.35	16 ± 2	8 ± 5	11 ± 4	71 ± 26	101 ± 125	106 ± 109	0.67 ± 0.32	0.41 ± 0.09	0.66 ± 0.13	300 ± 0	Highly Rated
SafeCig^b^	24	18.19 ± 0.16	-24.22	13 ± 0	14 ± 1	15 ± 2	68 ± 5	80 ± 11	93 ± 3	0.67 ± 0.09	0.72 ± 0.67	0.58 ± 0.24	300 ± 0	Highly Rated
LS Eagle	High	16.34 ± 0.13	NA	12 ± 2	9 ± 2	12 ± 4	59 ± 10	39 ± 15	68 ± 10	0.22 ± 0.12	0.36 ± 0.35	0.13 ± 0.07	172 ± 67	Not Rated
Smoke 51	16	25.72 ± 1.21	+60.75	4 ± 0	4 ± 0	4 ± 0	67 ± 24	85 ± 17	92 ± 15	0.39 ± 0.22	0.62 ± 0.13	0.36 ± 0.09	294 ± 11	Poorly Rated
SB Smoke	16	12.06 ± 0.20	-24.64	16 ± 1	14 ± 1	14 ± 1	45 ± 5	35 ± 5	42 ± 3	0.74 ± 0.31	0.70 ± 0.36	0.75 ± 0.23	300 ± 0	Top Five
V2 Cigs	18	15.61 ± 0.14	-13.28	12 ± 2	13 ± 0	13 ± 0	37 ± 8	45 ± 17	49 ± 15	0.86 ± 0.38	0.93 ± 0.08	0.87 ± 0.12	300 ± 0	Number One
BluCig	13–16	16.32 ± 0.24	+1.98	5 ± 2	9 ± 3	9 ± 3	14 ± 6	22 ± 9	22 ± 9	0.28 ± 0.29	0.63 ± 0.13	0.54 ± 0.15	300 ± 0	Highly Rated
Greensmoke	18	11.06 ± 0.17	-38.57	10 ± 0	9 ± 2	11 ± 2	64 ± 4	45 ± 11	74 ± 22	0.12 ± 0.09	0.56 ± 0.61	0.43 ± 0.34	233 ± 115	Top Five
Mark 10	2.5% weight	NA	NA	6 ± 3	7 ± 3	7 ± 3	23 ± 3	30 ± 22	33 ± 20	0.97 ± 0.05	0.79 ± 0.18	0.64 ± 0.10	268 ± 29	Major Tobacco
Vuse	4.8% weight	NA	NA	12 ± 2	12 ± 2	12 ± 2	49 ± 3	52 ± 4	55 ± 5	0.95 ± 0.24	0.94 ± 0.09	0.86 ± 0.05	214 ± 22	Major Tobacco

Abbreviations: 1st 10, average results from First 10 Puff protocol, 1st 10 –SO, average results from first 10 puffs during the smoke out, Smoke Out, average results from Modified Smoke-Out protocol, ND; Not Detected, NA; Not Applicable

^a^ Products from Williams, Talbot 2011 NTR

^b^ Products are available via third party vendors

^c^ Product was the number one selling brand at time of 2011 study

Greensmoke, BluCig, SafeCig, and South Beach Smoke were among the leading brands chosen by consumers (Table 1). V2 Cig was selected because the composition of the EC fluid was provided, and it was highly rated by consumers. Smoke 51 was a brand that was not highly rated. NJOY NPRO and Liberty Stix Eagle were chosen because we had evaluated the classic three piece counterparts in our prior studies [2]. Mark Ten and Vuse were chosen because they are produced by major tobacco companies. Upon receipt, all EC were inventoried and stored at room temperature until tested.

### Cartomizer Dissection and Fluid Separation

Fresh unused cartomizers were dissected to separate the fibers from the atomizing unit, as described previously [13]. The white plug in the end of the mouthpiece was removed to reveal the fibers surrounding the atomizing unit. The inner and outer fibers were centrifuged in MinElute Spin columns (Qiagen, Valencia, CA) at 14,000 revolutions/minute for 4–6 minutes to separate the fluid from the fibers [13].

### HPLC Analysis of Cartomizer Fluids

Samples of EC fluid were evaluated using a Hewlett Packard Series 1100 HPLC equipped with a 200 × 4.6 mm Thermo Scientific Hypersil ODS C18 column with a particle size of 5 μm. The 5% stock solutions of cartomizer fluid were made by dilution in a non-buffered mobile phase consisting of 77% water/ 23% acetonitrile (Fisher Scientific, Fair Lawn, NJ). Stock solutions were then diluted further in non-buffered mobile phase to the working concentration of 0.5%. The diode array detector signal was set to 260 nm with a bandwidth of 40 nm with a reference signal of 380 nm and bandwidth of 10 nm at a temperature of 35°C and a 0.8 ml/min flow rate. The mobile phase consisted of HPLC-grade chemicals (Fisher Scientific, Fair Lawn, NJ) in the following make up: 76.9% water, 23% acetonitrile, and 0.1% triethylamine; the pH of the mobile phase was adjusted daily to 7.6 using phosphoric acid (Fisher Scientific, Fair Lawn, NJ) and sodium hydroxide (EM Scientific, Gibbstown, NJ). The injection volume for all samples was 5 μl. The nicotine limit of quantification for this method was 10 μg/ml with a limit of detection of 50 ng/ml. The values reported are the means and standard deviations of the five runs. Full method details, including method validation, were published previously [5,7].

### Smoking Machine Set-Up

Experiments were done using a smoking machine that was connected through Tygon tubing to a water manometer, which is in turn was connected through Tygon tubing to a peristaltic pump [2,16,18–20]. EC puffs lasted 4.3 seconds and were taken every minute [21]. All cigarettes were smoked at the lowest airflow rate that produced a robust puff of aerosol. During each puff, pressure drop was measured using a water manometer. The aerosol was captured in a test tube every 10 puffs, and absorbance was measured in a spectrophotometer at a 420-nanometer reading [2,16]. Additionally, the airflow rate was calculated using the pump speed and a conversion factor provided by the pump manufacturer (Barnant Company, Barrington, IL).

### Performance Characteristics Experiments

#### First 10 Puff Protocol

Each EC was puffed 10 times with puffs spaced 1 minute apart. Pressure drop and air flow rate were recorded for each puff. Aerosol density was recorded for every other puff. Experiments were performed three times using a different EC cartomizers each time as described previously [2,16].

#### Smoke-out Protocol

To determine how air flow rate, pressure drop, and aerosol absorbance change during prolonged use, EC were puffed once every minute until cartridges were exhausted (pump speed reached its maximum and/or three consecutive puffs had aerosol densities below 0.05 absorbance units) or until 300 puffs were reached. Pressure drop and air flow rate were recorded for every puff, and aerosol absorbance was recorded every tenth puff. Air flow rate was increased by increasing pump speed by one interval on the pump dial whenever aerosol density dropped below 0.05 absorbance units or until pump speed reached its maximum air flow rate (24 mL/s) [2,16]. Three experiments were performed with each brand of EC. Each experiment used a different cartomizer. All cartomizers were fresh and had not been used previously by us. The lowest pump speed that produced robust aerosol was used for each brand. The pump was activated manually every minute, and pump speed was turned to zero between puffs to further resemble an active smoker. Pump speed was only increased if EC stopped producing aerosol.

## Results

### Appearance of EC

Cartomizer style EC come in different shapes, colors, and sizes. The 12 brands of cartomizer style EC that were used in this study are shown in Fig 1. Many manufacturers try to make their product resemble an actual cigarette (cig-a-like), although they are generally longer and heavier than conventional cigarettes. Most brands used in this study resembled conventional cigarettes.

### Performance Testing of Cartomizer Style EC

#### First 10 puffs protocol

**Fig 2:**
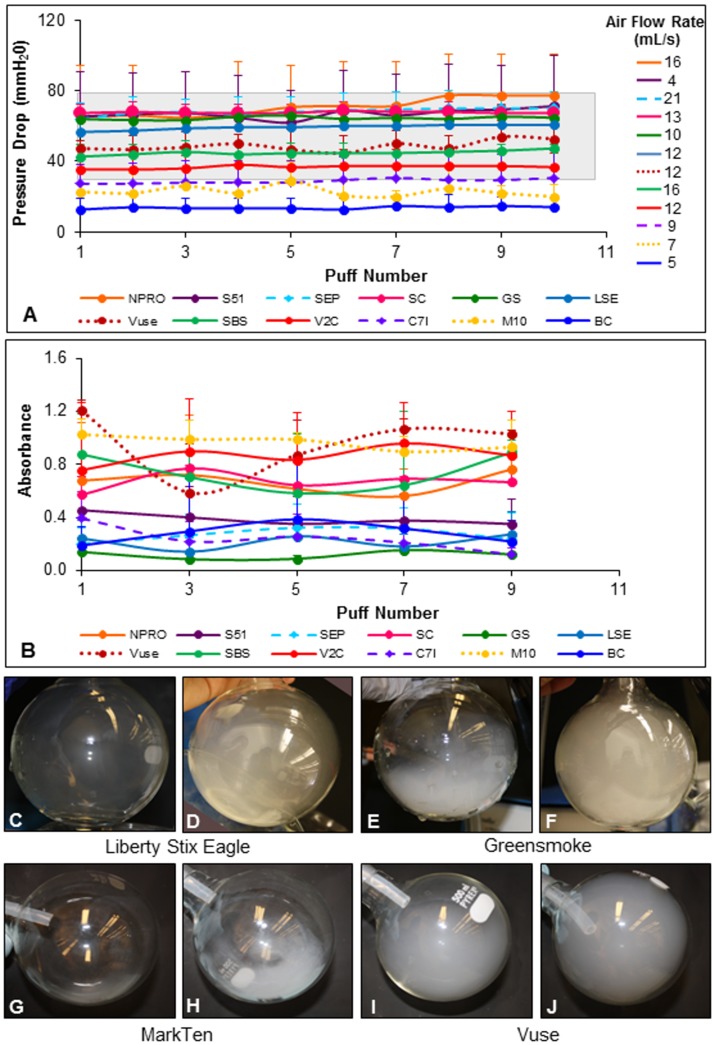
Pressure drop, airflow rate, and absorbance for the first 10 puffs. (A) Average pressure drop vs puff number for each brand. Air flow rates for each brand are listed in the legend on the right of the graph. The grey shaded box represents the pressure drop range for conventional cigarettes.[2,16] (B) Average absorbance vs puff number for EC over the first 9 puffs was similar within brands, but varied between brands. In A and B, each point is the mean ± standard deviation of three experiments. (C-J) Images of aerosol produced by two different cartomizers from Liberty Stix Eagle (C, D), Greensmoke (E, F). Mark Ten (G, H) and Vuse (I, J).

EC performance was compared among 12 brands for the first 10 puffs (Fig 2, Table 1). Pressure drop, which measures the leakiness of the EC to air during a puff, remained stable within a brand over the first 10 puffs, but varied between EC brands (Fig 2A). In contrast to the classic models of EC [2,16], most brands had pressure drops that were within the range of conventional cigarettes (~30–70 mm H_2_O), except for BluCig, Mark Ten, and Crown 7 Imperial, which were below this range (Fig 2A).

During the first 10 puffs, all EC required a single airflow rate to produce aerosol, and this rate, which ranged from 4–21 mL/s, did not change for any brand during puffing (Fig 2A, Table 1). In Trtchounian et al 2010, all conventional cigarettes required an air flow rate of 7 mL/s to produce smoke. Unlike conventional cigarettes, all EC brands, except three (Mark Ten, Smoke 51 and BluCig), required higher air flow rates than conventional cigarettes (Fig 2A, Table 1).

Aerosol absorbance, which is related to density, was measured spectrophotometrically over the first 10 puffs (Fig 2). The aerosol density was relatively stable for the first 10 puffs within a brand, but varied among brands (Fig 2B). Vuse and Mark Ten had the highest average absorbances (0.95± 0.24 and 0.97 ± 0.05, respectively) and Greensmoke had the lowest (0.12 ± 0.09) (Fig 2B, Table 1). For some products, aerosol density varied between cartomizers within a brand as shown in Fig 2C–2J. Two Liberty Stix Eagle (Fig 2C and 2D), two Greensmoke (Fig 2E and 2F), two Mark Ten (Fig 2G and 2H) and two Vuse (Fig 2I and 2J) cartomizers produced aerosol with very different densities within each brand. This variation in aerosol density within brands could contribute to the high standard deviations in absorbance readings observed for some brands (Fig 2B).

#### Modified Smoke-out Protocol

**Fig 3:**
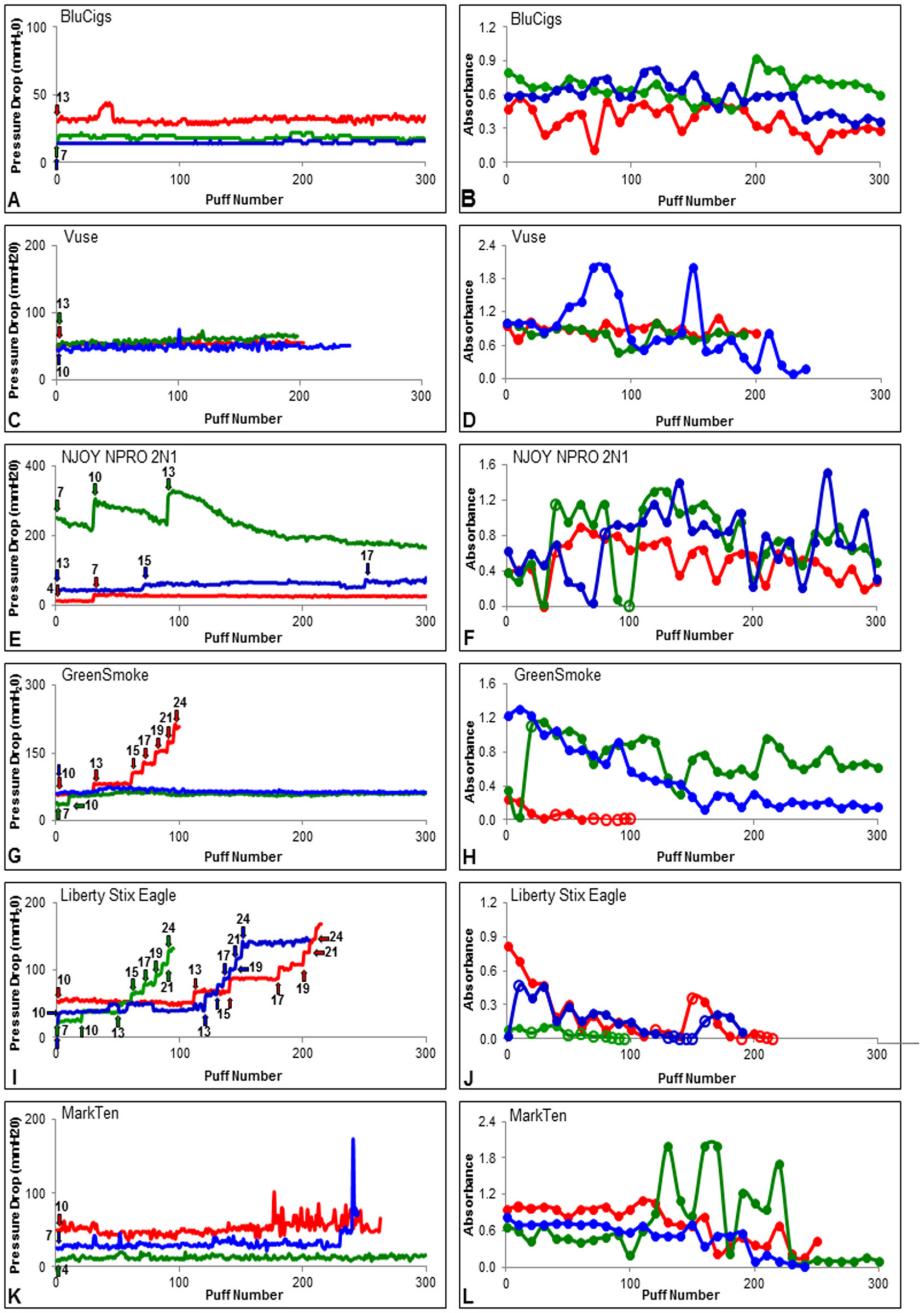
Results from the smoke-out protocol for six EC. In A, C, E, G, I, and K, pressure drop is plotted versus puff number for six brands. Arrows in A, C, E, G, I, and K indicate starting airflow rates (ml/s) and increases in airflow rate that were needed to continue aerosol production. In B, D, F, H, J, and L, absorbance is plotted versus puff number for the same six brands. Open circles indicate puffs where airflow rate was increased to maintain aerosol production. Data are shown for three different cartomizers for each brand. Trial 1 = red, trial 2 = green, and trial 3 = blue.

EC pressure drop, air flow rate required for aerosol production, aerosol absorbance, and puff number were evaluated by puffing cartomizers until either aerosol was no longer produced or 300 puffs were taken (Fig 3, S1 Fig, and Table 1).

Pressure drop data for six brands of EC are shown in (Fig 3A, 3C, 3E, 3G, 3I and 3K), and data for four additional brands are in (S1A, S1C, S1E, and S1G Fig). For each brand, three different fresh cartomizers were compared. Within brands, three distinct patterns of data were observed: (1) all three cartomizers performed similarly or the same, (2) two cartomizers were similar, while the third performed differently, and (3) all three cartomizers performed differently. The first performance pattern (all similar) was observed for BluCig, Vuse, Safe Cig, Smoke 51, South Beach Smoke, and V2 Cigs (Figs 3A, 3C, S1A, S1C, S1E and S1G). While occasionally a few puffs varied within a trial, most puffs were similar for a given cartomizer throughout the entire smoke out (Fig 3A and 3C and S1A, S1C, S1E and S1G Fig, Table 1). The second pattern (two similar and one different cartomizer) was seen in NJOY NPRO and Greensmoke (Fig 3E and 3G). The pressure drop for NJOY trials 1 and 3 (red and blue lines) were very similar, while trial 2 (green line) differed (Fig 3E). In trial 2, the pressure drop decreased, then peaked at puff 30 (300 mmH_2_O), decreased again, then peaked at puff 90 (300 mmH_2_0), then steadily decreased until puff 300 (Fig 3E). The Greensmoke cartomizers for trials 2 and 3 (green and blue lines in Fig 3G) performed similarly, while trial 1 (red line in Fig 3G) was clearly different. In Greensmoke trial 1 (red line in Fig 3G), the EC required repeated increases in air flow rate to produce aerosol, and this was accompanied by a corresponding increase in pressure drop. The pressure drop from trial 1 had steady increases starting at ~ puff 50 (Fig 3G). The third pattern was observed for Liberty Stix Eagle and Mark Ten in which the data from three trials were different from each other (Fig 3I and 3K). Each Liberty Stix Eagle cartomizer required increases in air flow rate in order to maintain aerosol production, and these increases were accompanied by corresponding increases in pressure drop (Fig 3I). In contrast for Mark Ten remained fairly constant through the smoke-out, but the three cartomizers had different pressure drops (Fig 3K)

The air flow rate was measured for every puff during the smoke out protocol and the initial airflow rates and any increases are indicated by arrows (Fig 3, S1 Fig). Smoke 51 and V2 Cigs used the same air flow rate (arrows) for all three trials (4 mL/s and 13 mL/s) for all 300 puffs (S1C and S1G Fig). For South Beach Smoke, two of the three cartomizers used a single air flow rate throughout the entire trial (15 mL/s), while the third cartomizer required an increase in the air flow rate to continue aerosol production (S1E Fig). For BluCig and Vuse and Mark Ten, all three cartomizers required a single air flow rate (arrows) throughout their trials, although the airflow rates varied within a brand (Fig 3A, 3C and 3K). For NJOY NPRO, each cartomizer used a different initial air flow rate (arrows), and each required increases throughout the 300 puffs (Fig 3E). For Greensmoke, the cartomizers in trials 2 and 3 required a single air flow rate (arrows) throughout the entire trial (10 mL/s), whereas the cartomizer in trial 1 required frequent increases in air flow rate to continue aerosol production (Fig 3G). The three cartomizers from Liberty Stix Eagle all required frequent increases in air flow rate to sustain aerosol production (Fig 3I).

The aerosol absorbance varied from puff to puff within brands as well as between brands (Fig 3, S1 Fig and Table 1). For South Beach Smoke, two trials were very similar, while the third trial had the same absorbance pattern, but produced less aerosol. The average yield for the three absorbance smoke-out trials was 0.75 ± 0.23 (S1F Fig, Table 1). Within the BluCig, Smoke 51, and V2 Cig groups, absorbance was similar for each trial with average absorbances of 0.54 ± 0.15, 0.36 ± 0.09, and 0.87 ± 0.12, respectively (Figs 3A, S1D and S1H, Table 1). For Greensmoke and SafeCig, absorbance decreased throughout the smoke-out (Fig 3G, S1B Fig). All three trials for NJOY NPRO produced significant amounts of aerosol, but the trials were not very similar (Fig 3F). All trials for Liberty Stix Eagle and one trial for Greensmoke did not produce a lot of aerosol, and thus required more frequent increases in air flow rate (Fig 3H and 3J). For SafeCig, the three trials all produced different amounts of aerosol in the beginning but towards the end of the smoke-out, the results were similar (S1B Fig). For both Mark Ten and Vuse, two cartomizers within groups produced similar aerosol, while the third was in each group was variable (Fig 3D and 3L).

All products except Vuse, Liberty Stix Eagle, and 2 of 3 Mark Ten cartomizers could be smoked up to 300 puffs (Fig 3D, 3J and 3L, Table 1). Greensmoke trial 1 (red line) stopped producing aerosol at puff 100, while the other two cartomizers produced 300 puffs (Fig 3H). The three trials for Liberty Stix Eagle did not last longer than 200 puffs (Fig 3J, Table 1).

The first 10 puffs from Fig 2 were compared to the first 10 puffs from the smoke-out (Fig 3 and S1 Fig) to determine how much variability there would be between two experiments done at different times with products purchased at different times (Table 1). Five of the brands (NJOY, Liberty Stix Eagle, Smoke 51, BluCig, and Greensmoke) produced quite different performance characteristics when comparing the data from the first 10 puff experiment to the first 10 puffs in the smoke-out experiment. As an example, comparisons for these two experiments for NJOY are: air flow rate: 16 and 8 ml/sec; pressure drop 71 and 101 mm H_2_O; and 0.67 and 0.41 absorbance units.

#### Nicotine Concentrations in Cartomizer Style Brands

Nicotine concentrations were determined in the cartomizer fluid from each sample evaluated in the performance trials (Table 1). Of 10 brands analyzed, only BluCig had a measured nicotine concentration within 10% of the value given on the manufacturer’s label. Most brands had less nicotine than the label indicated, and one brand (Smoke 51) had 60% more nicotine than indicated on the label.

## Discussion

The performance characteristics of 12 brands of cartomizer style EC were compared using short and long puffing protocols, and nicotine concentrations on labels vs measured concentrations were compared for each product that contained nicotine. Although cartomizer style EC are designed similarly, performance characteristics, such as air flow rate, pressure drop, and aerosol density varied among brands, which is consistent with our previous data [2,16]. Some of the cartomizer products performed similarly within brands (e.g. BluCig, Smoke 51, and V2 Cigs), while others did not (e.g. NJOY NPRO, Greensmoke, and Liberty Stix Eagle). In addition, some products performed differently when purchased at different times.

**Fig 4:**
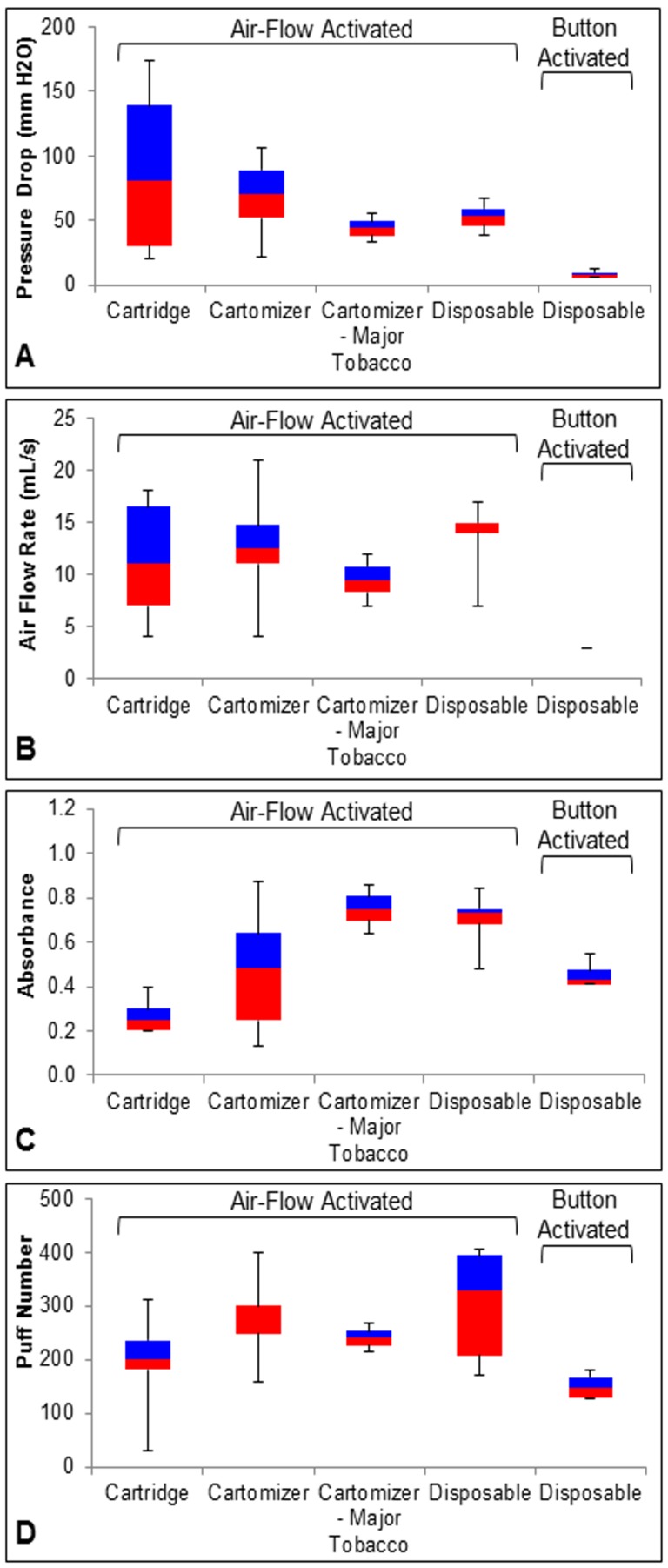
Comparison of performance properties across different styles of EC. Four performance properties, pressure drop (A), air flow rate (B), absorbance (C), and puff number (D), are summarized in box-whisker plots for cartridge models [2]; cartomizer models from our previous (16) and current study; disposable button-activated models [20]; disposable airflow activated models [20]. Each box shows the median, 75% percentile (blue), 25% percentile (red), and minimum and maximum values. The number of brands in each group was: six cartridge style, 10 cartomizer style, two major tobacco, five airflow-activated disposable brands, and four button-activated disposable brands.

Fig 4 summarizes and compares performance properties across four styles of EC, and S1 Table summarizes data for individual brands collected in this and our earlier studies. As mentioned earlier, pressure drop relates to the leakiness of the EC to air during a puff, and the lower the pressure drop the easier it is to draw air into the EC and produce aerosol. For most cartomizers in the current study, pressure drop was relatively stable during prolonged use, unlike the first generation classic cartridge models which had variable pressure drops (Fig 4A) [2,16]. Pressure drop for cartomizer EC ranged from 30 mm H_2_O to 100 mm H_2_O (Fig 4A). Cartomizers (Vuse and Mark Ten) from two major tobacco companies as well as the two disposable styles (button activated and air flow activated) had relatively low and uniform pressure drops both between and within brands [20]. The button activated models were interesting in that they had lower pressure drops than any of the other styles. As these devices have evolved, it appears pressure drop has become more uniform within a style and pressure drop values have become similar to those of conventional cigarettes [2].

The various styles of EC required different air flow rates for aerosol production (Fig 4B). Cartridge models were highly variable in the air flow rate required for activation and also required frequent increases in air flow rate during the smoke-out protocol for continued aerosol production [2,16]. Cartomizer style EC were activated by a broad range of air flow rates, with most brands producing aerosol between 4–21 mL/s (Fig 4B) [2,16]. Air flow rate requirements for activation were very similar in the major tobacco group. Button-activated disposable models all used the same air flow rate for activation. All air flow activated models required between 14–17 mL/s, except for BluCigs which were activated by 7 mL/s [20]. The evolution of EC products towards lower air flow rates for activation may be a benefit for users.

In our previous performance studies, aerosol absorbance, which is a measure of aerosol density, was quite uniform within each group of EC [2,16,20]. In contrast, the aerosol absorbance for the cartomizer models in this study (excluding major tobacco) was variable and ranged from 0.13 to 0.87 average absorbance units/smoke-out (Table 1, Fig 4C). This range was greater than for any of the other groups (Fig 4). Within the cartomizer group, absorbance for each brand differed with some brands producing fairly uniform aerosol between cartomizers, while others did not. The cartomizers from the major tobacco companies and the air flow activated models produced aerosol with the highest densities. The variability in aerosol absorbance in the in some brands in the cartomizer group indicates a need for better quality control in the manufacturing of these devices.

While puff number in the major tobacco and button activated models were very uniform within groups, puff number varied in the other three categories (Fig 4D). Puff number was highest in the air activated style EC and lowest in the button activated. Cartridge style EC lasted for a wide range of puffs, as few as 25 to as many as 300 puffs. Except for Smoking Everywhere Platinum, Liberty Stix Eagle, Greensmoke, Vuse, and Mark Ten, cartomizer style EC lasted for 300 puffs or more, which is longer than the often advertised puff number (one cartomizer = about 1 package of conventional cigarettes or 200 puffs according to most/some/all advertisements) (Fig 4D). Vuse advertises that their brand will last about 200 puffs, and all units we tested lasted at least this long. Button-activated EC never lasted longer than 200 puffs. While the air-flow activated models varied in the number of puffs, most models lasted less than 300 puffs. None of the disposable brands lasted their advertised number of puffs, which could be attributed to the battery. In most cases, disposable units stopped producing aerosol because the battery, which is not rechargeable, died. It is not known how long the disposable units sit in warehouses and in shops before use, but most have probably lost some of their charge before purchase [20].

There were also discrepancies in the labeling of nicotine concentrations on EC packages, as reported previously for other EC products [7,22,23]. Only one brand, BluCig, met the American E-Liquid Manufacturing Standards Association (AEMSA) standard for nicotine labeling which requires that the measured and labeled concentration deviate by less than 10%. Most cartomizer brands contained less nicotine than the label on the cartons indicated, although one brand (Smoke 51) had 60% more than the labeled concentration. These labeling discrepancies are in agreement with a recent study that measured the amount of nicotine in refill fluids and found that 35 out of 54 products had nicotine concentrations that deviated from the labeled concentration by more than 10% [7]. Two brands (Liberty Stix Eagle and Crown 7 Imperial) did not give a nicotine concentration, but ranked nicotine as low, medium, and high, or bold. Proper nicotine labeling is a public health concern. Some EC refill bottles without any label contained over 100 mg/ml of nicotine [7], and some do-it-yourself flavor products that are presumed to be nicotine free contained nicotine [24]. The variations in performance parameters and discrepancies in nicotine concentrations may help understand the variability in consumer puffing patterns and why EC users take more puffs, longer puffs, and more frequent puffs [21,25,26].

In summary, performance parameters were generally more consistent in cartomizer style EC than in the classic cartridge style, (except for aerosol absorbance which was most variable in the cartomizer group), indicating an improvement in performance with the evolution of these products. However, for 5 of the brands there was considerable variation in products purchased at different times. Of the four classes of EC that we have studied, major tobacco cartomizers and button-activated disposable brands were the most uniform for all performance parameters across and within brands; however, puff number for button-activated models was lower than advertised and lower than any of the other groups. For the cartomizer style EC in the 10 puff protocol, there was little variation within brands, but significant variation between brands. In the smoke-out protocol, most cartomizer brands had relatively stable pressure drop, air flow rate, and absorbance, while a few cartomizers behaved differently than others in their group. The highly variable aerosol absorbances observed in the cartomizer group, the variation in performance parameters within some brands in the cartomizer group, the low puff numbers achieved with disposable brands, and the variation in performance for some products purchased at different times indicate a need for better quality control in the manufacture and design of EC.

## Supporting Information

S1 Fig Results from the smoke-out protocol for four EC brands. (A, C, E, and G) Pressure drop is plotted versus puff number for SafeCig, Smoke 51, South Bach Smoke, and V2 Cigs. Arrows in A, C, E and G) indicate starting airflow rates (ml/s) and increases in airflow rate that were needed to continue aerosol production. (B, D, F and H) Absorbance is plotted versus puff number for the same brands. Open circles indicate puffs where airflow rate (pump speed) was increased to maintain aerosol production. Data are shown from three different cartomizers for each brand. Trial 1 = red, trial 2 = green, and trial 3 = blue.
(PDF)

S1 Table Consolidation of Performance Parameters for all EC devices. (DOCX)
