# Supplementary figures and images for: Comparison of the Performance of Cartomizer Style Electronic Cigarettes from Major Tobacco and Independent Manufacturers
Source: PLoS One. 2016 Feb 18;11(2):e0149251. doi: 10.1371/journal.pone.0149251 (PMC4758646; doi:10.1371/journal.pone.0149251)

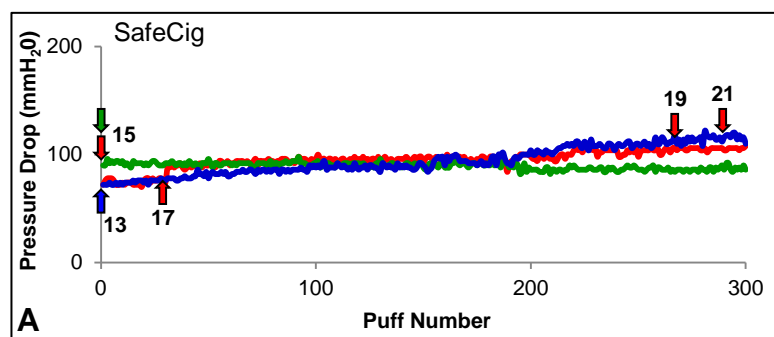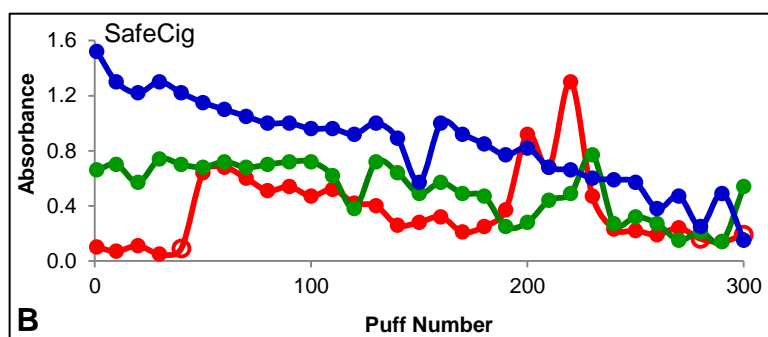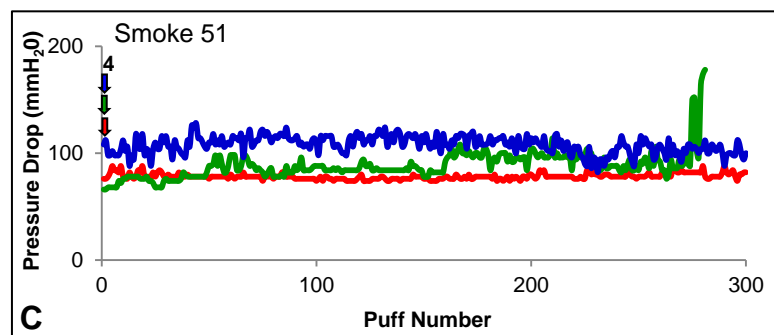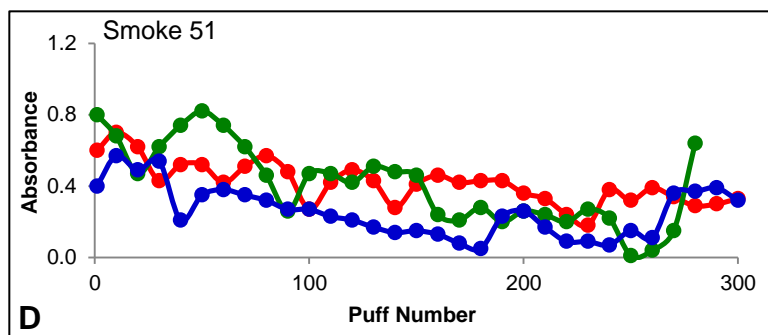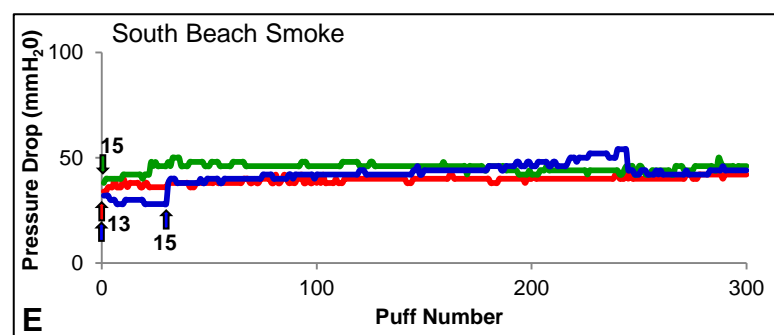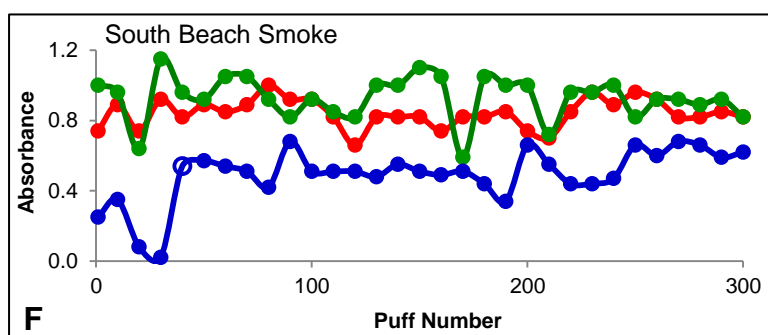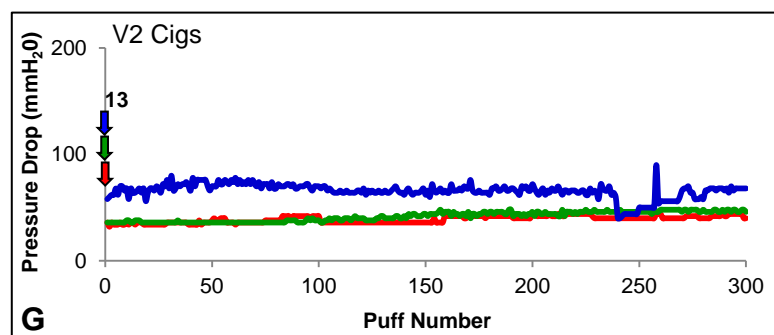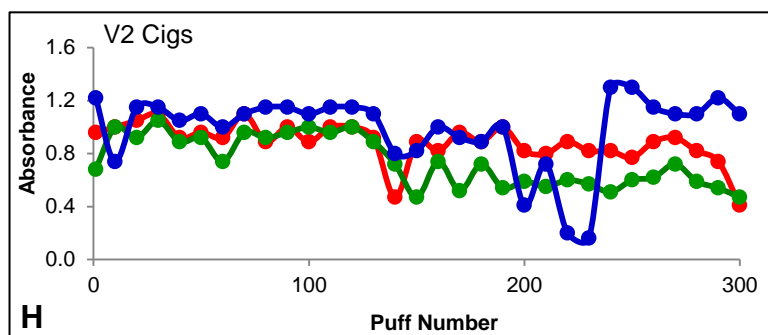

Supplemental Figure 1

Supplement: S1 Fig — (A, C, E, and G) Pressure drop is plotted versus puff number for SafeCig, Smoke 51, South Bach Smoke, and V2 Cigs. Arrows in A, C, E and G) indicate starting airflow rates (ml/s) and increases in airflow rate that were needed to continue aerosol production. (B, D, F and H) Absorbance is plotted versus puff number for the same brands. Open circles indicate puffs where airflow rate (pump speed) was increased to maintain aerosol production. Data are shown from three different cartomizers for each brand. Trial 1 = red, trial 2 = green, and trial 3 = blue. (PDF) [file pone.0149251.s001.pdf]
