# Supplementary material for: Comparison of the Performance of Cartomizer Style Electronic Cigarettes from Major Tobacco and Independent Manufacturers
Source: PLoS One. 2016 Feb 18;11(2):e0149251. doi: 10.1371/journal.pone.0149251 (PMC4758646; doi:10.1371/journal.pone.0149251)
Supplement: S1 Table — (DOCX) [file pone.0149251.s002.docx]

| **Brand** | **Average**  **Air Flow Rate (mL/s)** | **Average**  **Pressure Drop (mmH_2_O)** | **Average**  **Absorbance** | **Average**  **Puff Number** |
| --- | --- | --- | --- | --- |
| Cartridge | | | | |
| SE Gold^a^ | 18 ± 1 | 158 ± 26 | 0.20 ± 0.20 | 177 ± 15 |
| NJOY NCIG^a^ | 21 ± 1 | 128 ± 18 | 0.20 ± 0.10 | 313 ± 115 |
| Crown 7 Hydro^a^ | 16 ± 1 | 174 ± 23 | 0.30 ± 0.20 | 208 ± 34 |
| Liberty Stix^a^ | 11 ± 0 | 34 ± 6 | 0.40 ± 0.30 | 197 ± 64 |
| VapCigs^a^ | 12 ± 2 | 21 ± 2 | 0.30 ± 0.20 | 30 ± 43 |
| VapCigs 2^b^ | 6 ± 0 | 29 ± 7 | 0.20 ± 0.04 | 245 ± 18 |
| Cartomizers – Independent manufacturers | | | | |
| SE Platinum^b^ | 21 ± 1 | 79 ± 5 | 0.16 ± 0.05 | 160 ± 66 |
| C7 Imperial^b^ | 15 ± 1 | 62 ± 8 | 0.21 ± 0.06 | 400 ± 10 |
| NJOY NPRO | 11 ± 4 | 106 ± 109 | 0.66 ± 0.13 | 300 ± 0 |
| SafeCig | 15 ± 2 | 93 ± 3 | 0.58 ± 0.24 | 300 ± 0 |
| LS Eagle | 12 ± 4 | 68 ± 10 | 0.13 ± 0.07 | 172 ± 67 |
| Smoke 51 | 4 ± 0 | 92 ± 15 | 0.36 ± 0.09 | 294 ± 11 |
| SB Smoke | 14 ± 1 | 42 ± 3 | 0.75 ± 0.23 | 300 ± 0 |
| V2 Cigs | 13 ± 0 | 49 ± 15 | 0.87 ± 0.12 | 300 ± 0 |
| BluCig | 9 ± 3 | 22 ± 9 | 0.54 ± 0.15 | 300 ± 0 |
| Greensmoke | 11 ± 2 | 74 ± 22 | 0.43 ± 0.34 | 233 ± 115 |
| Cartomizer – Major Tobacco | | | | |
| Mark 10 | 7 ± 3 | 33 ± 20 | 0.64 ± 0.10 | 268 ± 29 |
| Vuse | 12 ± 2 | 55 ± 5 | 0.86 ± 0.05 | 214 ± 22 |
| Disposable – Air Flow Activated | | | | |
| BluCig^c^ | 7 ± 0 | 39 ± 5 | 0.48 ± 0.11 | 331 ± 13 |
| NJOY King^c^ | 15 ± 5 | 67 ± 34 | 0.75 ± 0.28 | 171 ± 37 |
| V2 Cigs^c^ | 14 ± 1 | 59 ± 5 | 0.73 ± 0.13 | 208 ± 47 |
| Smooth^c^ | 15 ± 0 | 46 ± 8 | 0.84 ± 0.02 |  |
| Tsunami^c^ | 17 ± 0 | 54 ± 2 | 0.68 ± 0.25 |  |
| Disposable – Button Activated | | | | |
| Square 82^c^ | 3 ± 0 | 6 ± 4 | 0.41 ± 0.07 | 126 ± 25 |
| Imperial Hookah^c^ | 3 ± 0 | 12 ± 0 | 0.55 ± 0.11 | 163 ± 12 |
| Luxury Lights^c^ | 3 ± 0 | 9 ± 5 | 0.41 ± 0.14 | 182 ± 19 |
| Starbuzz^c^ | 3 ± 1 | 6 ± 7 | 0.45 ± 0.09 | 130 ± 2 |
| ^a^Products from Trtchounian, Williams, Talbot 2010 NTR | | | | |
| ^b^Products from Williams, Talbot 2011 NTR | | | | |
| ^c^Products from Williams, Ghai, Talbot 2014 NTR | | | | |

Supplementary Table 1: Performance properties by brand for cartridge, cartomizer and disposable EC
